# Diagnostic Challenges and Multidisciplinary Management of Invasive Mediastinal Adenocarcinoma: A Case Report

**DOI:** 10.7759/cureus.84995

**Published:** 2025-05-28

**Authors:** Yasushi Sakamaki, Naoya Takada, Hiromi Tsuji, Yuya Kogita, Nanami Hiraiwa

**Affiliations:** 1 Department of Thoracic Surgery, Osaka Keisatsu Hospital, Osaka, JPN; 2 Department of General Thoracic Surgery, Osaka General Medical Center, Osaka, JPN; 3 Department of Diagnostic Pathology, Osaka Rosai Hospital, Sakai, JPN; 4 Department of General Thoracic Surgery, Osaka Toneyama Medical Center, Toyonaka, JPN

**Keywords:** mediastinal adenocarcinoma, mediastinum malignancy, metastatic adenocarcinoma of unknown primary, metastatic cancer of unknown primary, metastatic lymph node carcinoma

## Abstract

Adenocarcinoma rarely manifests as a bulky, invasive mediastinal tumor. Even more unusual is when such a tumor, initially diagnosed as another malignancy on biopsy, is later identified as an adenocarcinoma of unknown primary origin.

A 46-year-old man with no prior history of malignancy was diagnosed with primary mediastinal seminoma and received chemotherapy before being referred to our hospital. At referral, the tumor had shrunk to half its original size, measuring 7 cm in maximum diameter. Due to its extensive invasion, we performed a combined resection, including the resection and reconstruction of the superior vena cava, achieving complete tumor removal. However, the resected specimen showed extensive necrosis, making it unidentifiable as a tumor. This prompted a reexamination of the pretreatment biopsy, which led to a revised diagnosis of “adenocarcinoma not otherwise specified with clear cell features.” Immunohistochemical staining suggested a lung cancer profile, but systemic examination failed to identify the primary site, resulting in a final diagnosis of carcinoma of unknown primary. The cancer recurred 26 months after surgery, and the patient underwent repeated chemotherapy up to the fourth line. He survived for 92 months after surgery before passing away.

This case highlights the diagnostic challenges posed by invasive mediastinal adenocarcinoma of unknown primary. It also demonstrates that multidisciplinary treatment - combining induction chemotherapy, curative surgery, and post-recurrence chemotherapy - can enable long-term survival, even in such complex cases.

## Introduction

Adenocarcinomas, unlike other malignancies such as lymphomas, rarely present as large invasive tumors in the mediastinum [1]. We report a case of a bulky mediastinal tumor, initially diagnosed as extragonadal seminoma via biopsy and treated with chemotherapy followed by curative surgery, which was ultimately identified as an adenocarcinoma of unknown primary origin after significant challenges in establishing the final histological diagnosis.

## Case presentation

Characteristics

**Figure 1:**
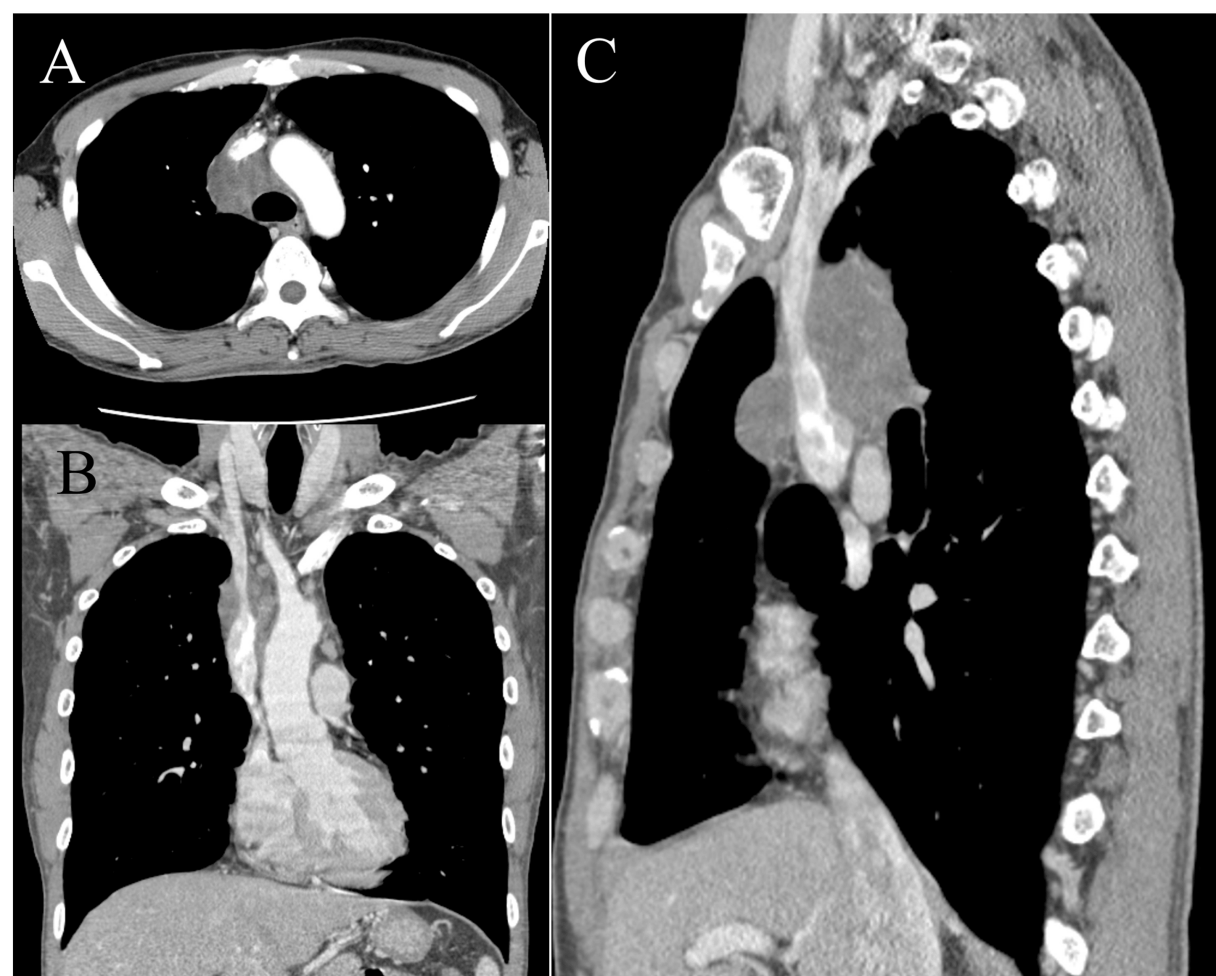
Mediastinal tumor after neoadjuvant chemotherapy (A) Axial, (B) coronal, and (C) sagittal views of computed tomography show the tumor reduced to 7 cm in maximum dimension after chemotherapy. Primarily located in the right lower paratracheal region, it extensively invaded the superior vena cava, especially posteriorly. (Pretreatment images unavailable due to expired storage period.)

A 46-year-old male smoker with no prior history of malignancies was referred to our department for definitive surgery within two months of his last chemotherapy session at the referring hospital (RH). Initially diagnosed with primary mediastinal seminoma via biopsy, he had received three of the four planned courses of triplet therapy (bleomycin, etoposide, and cisplatin, i.e., BEP) before referral. At the time of referral, the tumor-though still large-had shrunk to 7 cm in maximum diameter, half its pretreatment size, as shown by computed tomography (Figure 1).

The referral was prompted by the tumor’s extensive invasion into the superior vena cava (SVC), necessitating a hospital with thoracic and cardiovascular surgeons capable of SVC reconstruction concomitant with extensive tumor resection. However, the biopsy’s histologic diagnosis of seminoma was considered presumptive, as immunohistochemical staining (IHC) showed atypical results for a germ cell tumor, with positive expression of epithelial membrane antigen (EMA) and carcinoembryonic antigen (CEA). A metastatic workup revealed no other lesions, and tumor markers, including beta-human chorionic gonadotropin, remained consistently negative before and after BEP therapy.

Surgery

**Figure 2:**
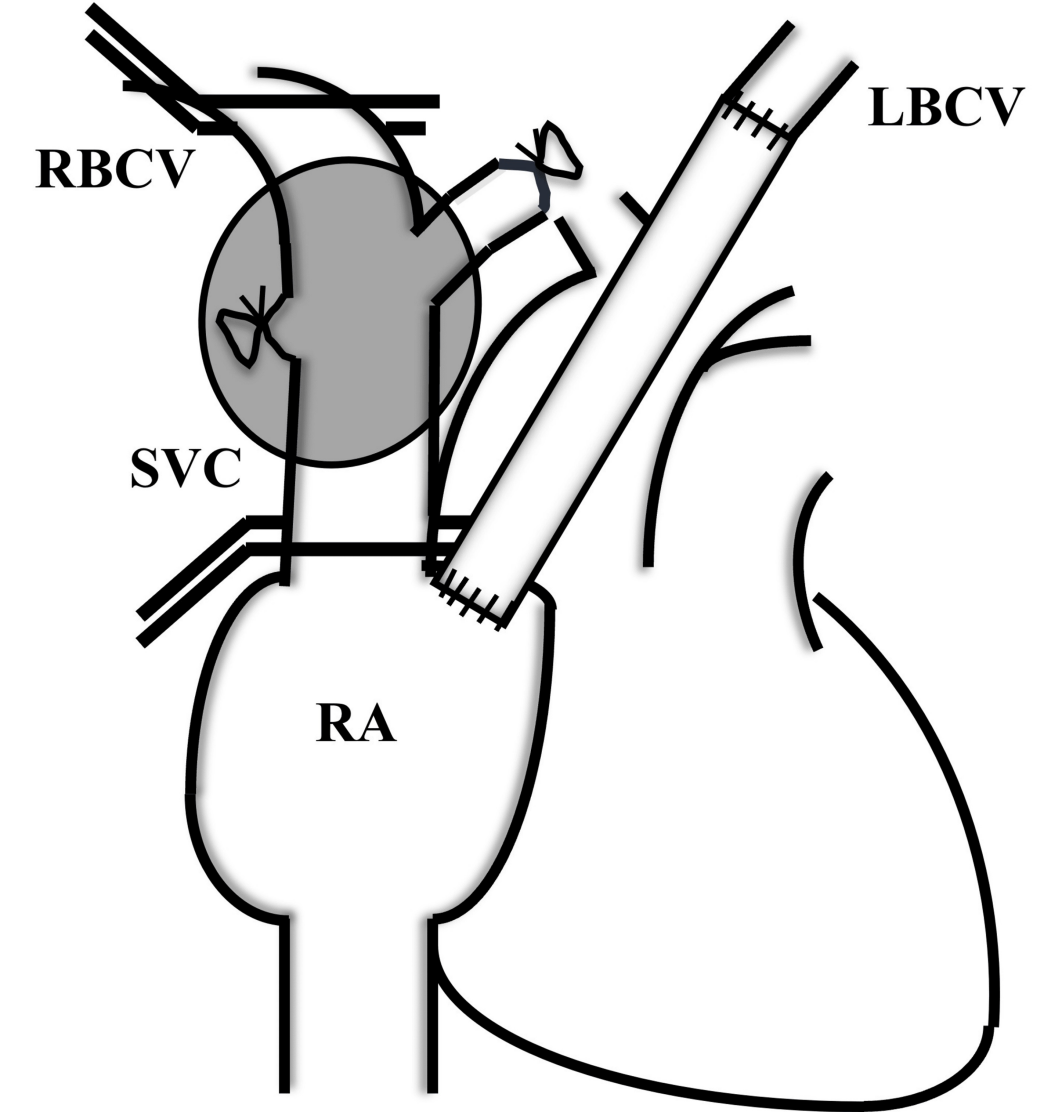
Surgical findings and reconstruction schema SVC: superior vena cava; RA: right atrium; LBCV: left brachiocephalic vein; RBCV: right brachiocephalic vein. The gray circle represents the tumor. This image is drawn by the authors of this article.

Sixty days after the last administration of chemotherapy, we performed a complete tumor resection via median sternotomy. The tumor was primarily located in the right lower paratracheal region, with lesser involvement in the anterior mediastinum, and surgical findings did not support a thymic origin. The tumor was firmly attached to the trachea without infiltrating it, and the right lung appeared to be an infiltrated organ rather than the primary site. Due to extensive invasion, the surgery required simultaneous resection of the thymus, part of the right upper lobe, the right phrenic nerve, and the SVC. To reconstruct the vasculature, we sequentially anastomosed two vascular grafts between each brachiocephalic vein and the right atrium. The reconstruction involved two key steps: (1) connecting the left brachiocephalic vein to the right atrial appendage using an artificial graft, and (2) removing the occluded SVC by placing vascular clamps proximal and distal to the invasion site, then reconstructing the right brachiocephalic vein with another graft (Figure 2). This approach eliminated the need for cardiopulmonary bypass.

Pathology

**Figure 3:**
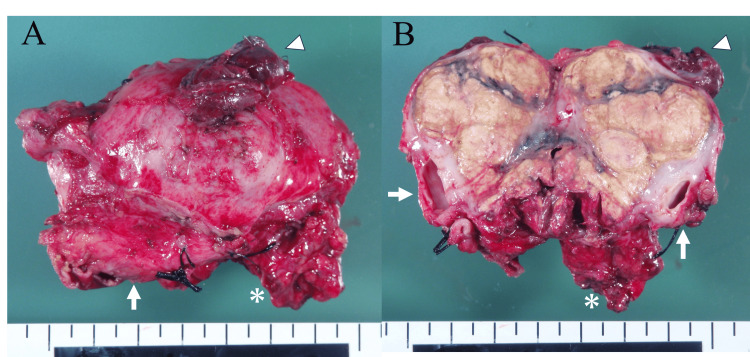
Resected specimen (A) Gross appearance of the resected tumor. (B) Cut surface showing the superior vena cava (arrows), lung (arrowheads), and thymus (asterisks), oriented with right as cranial and left as caudal.

Although the resected tumor appeared largely viable (Figure 3).

**Figure 4:**
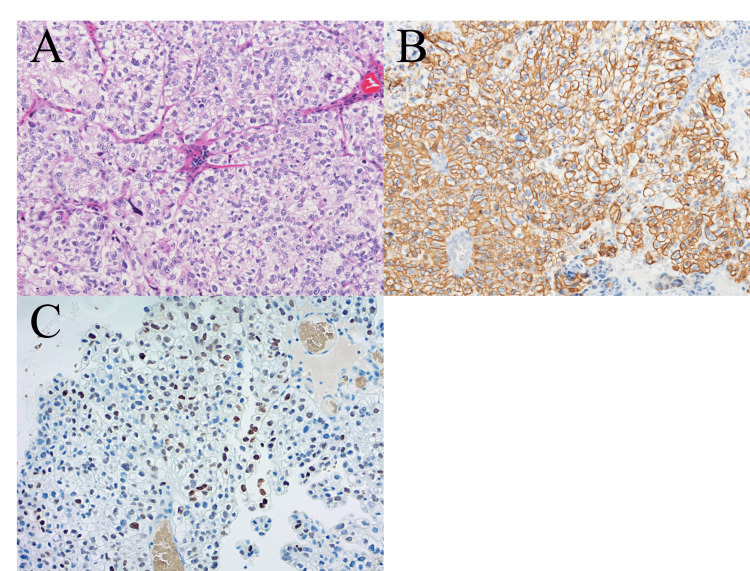
Pretreatment histology and immunohistochemistry (A) Hematoxylin–eosin staining shows atypical cells with large nuclei and clear cytoplasm. Immunoreactivity includes: (B) Cytokeratin 7 and (C) Thyroid transcription factor 1.

Histological examination revealed extensive necrosis, rendering it nearly impossible to identify as a tumor. This prompted a reexamination of the pretreatment biopsy specimen (provided as unstained thin sections by the RH), which showed a homogeneous composition of cells with clear cytoplasm and large, atypical nuclei (Figure 4A).

**Figure 5:**
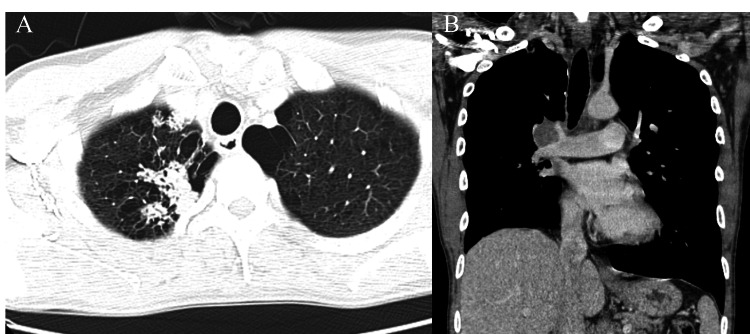
Recurrence sites on computed tomography At the time of first recurrence (26 months after surgery). (A) Multiple lesions in the right upper lobe, (B) Right hilar lymph node.

Initial IHC and histology led to differential diagnoses that included not only seminoma but also carcinoma with clear cell features, with the latter considered more likely and reported to the RH. However, the final diagnosis of “adenocarcinoma, not otherwise specified, with clear cell features” was established years later through a case review and personal communication with an expert who analyzed both histological and IHC findings. Subsequent IHC provided critical insights: positivity for cytokeratin 7 and thyroid transcription factor 1 (TTF-1) suggested a pulmonary epithelial origin (Figures 4B-4C). The tumor cells also showed positivity for placental alkaline phosphatase (PLAP), class III receptor tyrosine kinase (c-kit), EMA, and CEA. However, the positivity for EMA and CEA, combined with negativity for spalt-like transcription factor 4 (SALL4) - a highly sensitive marker for germ cell tumors - ruled out seminoma definitively. Additionally, negativity for CD5 excluded thymic carcinoma, consistent with surgical findings. Although IHC suggested a lung cancer profile, the CEA-positive tumor was classified as an adenocarcinoma of unknown primary site due to the absence of an identified primary lesion. It could not be categorized as an N2 lung cancer of unknown primary due to the absence of identifiable lymph node histology. Outcome. Following an uneventful recovery from surgery, the patient returned to the RH for regular follow-up to monitor for recurrence. Twenty-six months later, new lesions were detected in the right upper lobe and pulmonary hilar lymph node (Figure 5).

Transbronchial biopsy at our hospital histologically confirmed that the lung lesions shared key features, including adenocarcinoma morphology and IHC pattern, with pretreatment mediastinal tumor specimens, suggesting recurrence. After the recurrence was confirmed, the patient declined further surgery and instead received first-line chemotherapy (two courses of vinblastine, ifosfamide, and cisplatin triplet therapy) at the RH. At that time, the RH prioritized the initial seminoma diagnosis over carcinoma, as the final diagnosis remained inconclusive. After more than a year had passed, as the disease had progressed again, the patient requested transfer to our hospital, where he underwent repeated chemotherapy up to the fourth line (three courses of cisplatin plus docetaxel as the second line, two courses of carboplatin plus nanoparticle albumin-bound paclitaxel (nab-PTX) as the third line, and four courses of nab-PTX as the fourth line) and survived for over four years. The maximum effect of each chemotherapy was stable disease, which became progressive disease within about a year. He ultimately passed away at home 92 months after surgery, with no autopsy performed.

## Discussion

This case of mediastinal cancer of unknown primary (CUP) presented unique challenges. Initially, the preoperative diagnosis of seminoma conflicted with postoperative findings of adenocarcinoma, but extensive necrosis in the resected specimen precluded clarification. The pretreatment biopsy, however, confirmed seminoma, revealing discrepancies between our institution’s interpretation and the RH’s, despite analyzing the same biopsy. Remarkably, preoperative chemotherapy achieved a complete response, and the patient survived more than 5 years even after recurrence while receiving other chemotherapies - far exceeding the median survival for patients with CUP treated with chemotherapy alone [2, 3] - demonstrating effective clinical management despite early misdiagnosis. Yet, recurrence 26 months after complete resection defied expectations, underscoring that mediastinal CUP can relapse even after apparent eradication.

In this case, reexamination of the pretreatment biopsy specimen led to the diagnosis of an originally 14-cm mediastinal adenocarcinoma before BEP therapy (pretreatment images unavailable at manuscript preparation due to expired storage period), but neither a primary site nor lymph node origin was identified despite whole-body CT, endoscopy, and tumor marker analysis. Typically, mediastinal adenocarcinomas represent metastases to lymph nodes from primaries such as lung or colorectal cancer. However, in rare cases, the primary remains occult despite exhaustive workup [4-6]. Although mediastinal lymph node involvement accounts for only 1.5% of CUP cases [4, 5], of all metastatic adenocarcinomas in the mediastinal lymph nodes, those with an unknown primary site might conservatively account for approximately 1% to 4%, an estimated range of ours based on fragmented data from case reports and smaller studies [4-7]. Thymic adenocarcinoma, a candidate due to its mediastinal location, is particularly rare, with only about 30 cases reported to date [8], while ectopic primaries (e.g., parathyroid or pancreatic cancer) are even less common [9, 10]. Surgical findings suggested an origin in the right lower paratracheal lymph nodes, based on tumor proximity and absence of other primaries. Extensive necrosis obscured histological detail, but we hypothesize that less necrosis might have revealed residual lymph node architecture, supporting this origin. We previously reported another mediastinal CUP case treated with chemoradiotherapy and surgery (Table 1) [11], comparing the differences in diagnostic and therapeutic outcomes between the two cases.

**Table 1:** Characteristics of patients with mediastinal adenocarcinoma of unknown primary who underwent definitive surgery. Cases 1 and 2 refer to the reported case [11] and the present case, respectively. Age represents age (years) at surgery. Outcome includes the postoperative interval. Ad: adenocarcinoma; BEP: bleomycin + etoposide + cisplatin; CBDCA: carboplatin; CD: cluster of differentiation; CDDP: cisplatin; CEA: carcinoembryonic antigen; CK: cytokeratin; c-kit: class III receptor tyrosine kinase; DOD: died of disease; DTX: docetaxel; EMA: epithelial membrane antigen; IHC: immunohistochemical staining; nab-PTX: nanoparticle albumin-bound paclitaxel; NED: no evidence of disease; NOS: not otherwise specified; PLAP: placental alkaline phosphatase; RT: radiotherapy; SALL4: spalt-like transcription factor 4; TTF-1: thyroid transcription factor 1; VeIP: vinblastine + ifosfamide + cisplatin; VNR: vinorelbine. *Two-course administration with concurrent 40-Gy irradiation. ^+^Three-course administration ^+, #^Administered at the referring hospital.

Case	Age /sex	Pretreatment diagnosis	Postoperative diagnosis	IHC findings (reactivity)	Lymph node architecture	Preoperative treatment	Treatment for recurrence	Outcome (month)
1[11]	46/Male	Ad	Ad with neuroendocrine differentiation	CK7 (+), CK20 (−), TTF-1 (−), Napsin A (−), Synaptophysin (+), CD5 (−).	Identified	CDDP+VNR with RT^*^	Not applicable	NED (156)
2	46/Male	Seminoma	Ad, NOS, with clear cell features	CK7 (+), CK20 (−), TTF-1 (+), PLAP (+), c-kit (+), CEA (+), EMA (+), SALL4 (−), CD5 (−).	Unidentified	BEP^+^	VeIP^#^ CDDP+DTX CBDCA+nab-PTX nab-PTX	DOD (92)

Despite an initial misdiagnosis of the tumor as a germ cell malignancy, our patient experienced substantial clinical benefit from the treatment course. The preoperative BEP therapy, typically reserved for germ cell tumors rather than the correctly identified adenocarcinoma, reduced the tumor size by approximately 50% in maximum diameter and induced extensive necrosis, rendering it histologically unidentifiable as viable tumor tissue. This remarkable response facilitated complete surgical resection, which spared the patient from additional preoperative chemotherapy that might have been less effective given the tumor’s ultimate classification. Conversely, had the tumor been accurately diagnosed as adenocarcinoma from the outset, it is unclear whether a standard regimen, such as a platinum-based doublet (e.g., cisplatin plus pemetrexed), would have achieved a comparable downsizing effect, given that response rates for adenocarcinoma typically range from 20% to 40% with such regimens [12]. For context, this case aligns with CUP, specifically mediastinal CUP, which constitutes part of the 80% of CUP cases with poor prognosis [6]; these patients typically face a median overall survival of less than 12 months when treated with chemotherapy alone [2, 3]. Strikingly, our patient not only survived a recurrence but did so for more than 5 years thereafter, sustained by chemotherapy regimens detailed in Table 1, far exceeding the typical prognosis for unresectable CUP.

In this case, multiple pulmonary lesions emerged after surgery (Figure 5A) and were histologically confirmed by biopsy to indicate recurrence. However, the diagnostic complexity of CUP complicates their classification: these lesions might represent a direct recurrence of the mediastinal CUP or signal an occult lung primary that surfaced later, potentially accompanied by ipsilateral lymph node metastases, as described in prior reports [13]. Although no lymph node involvement was confirmed histologically within the initial tumor, the ambiguity persists without autopsy data, which could have identified a distinct lung primary through unique tumor architecture or marker expression. Regardless, the distribution of these lesions-spanning multiple lung segments with hilar lymph node involvement (Figure 5B), suggested tumor cell dissemination via both hematogenous and lymphatic routes, mirroring patterns seen in lung cancer with a known primary. Literature notes that some mediastinal CUP cases with lymph node involvement exhibit a lung cancer profile on IHC, such as TTF-1 positivity, and are staged as N2 lung cancer with an occult primary [4-6, 11, 13]. In our patient, IHC revealed similar lung-associated markers (e.g., TTF-1, CK7), though the absence of confirmed nodal metastases precluded an N2 classification. Based on these findings, we managed the recurrence as akin to lung cancer, employing regimens such as cisplatin plus docetaxel-detailed in Table 1-over most of the treatment period. Consequently, this approach aligned with standard protocols for lung cancer with a known primary [14], a logical choice given the overlapping IHC and behavioral profiles.

When our patient was treated in the 2010s, the standard approach for poor-prognosis CUP relied primarily on nonspecific platinum-based regimens as supported by contemporary guidelines [2, 3]. Since that time, advancements in the 2020s - most notably the adoption of next-generation sequencing - have enabled clinicians to infer primary tumor origins in CUP cases through tissue-of-origin classifiers and identify molecularly guided therapy options, such as targeted agents or immunotherapy [15]. For instance, a prospective randomized phase II trial recently showed that patients receiving molecularly guided therapy, predominantly atezolizumab for immune-responsive profiles, achieved a median progression-free survival of 6.1 months, compared to 4.4 months with standard platinum-based chemotherapy alone [16]. Looking forward, molecularly guided therapy, powered by comprehensive genomic profiling, is poised to further refine CUP management, potentially doubling overall survival rates as profiling accuracy improves. In this evolving landscape, curative surgery for poor-prognosis CUP might emerge as a viable option for selected patients-particularly those whose tumors are localized and downsized by personalized therapies-offering a chance at long-term control where systemic approaches alone fall short, especially due to variable response rates and limited curative potential.

## Conclusions

We treated a patient with an invasive, bulky mediastinal adenocarcinoma of unknown primary, which posed diagnostic challenges. This case also demonstrates that multidisciplinary treatment, especially combining radical surgery after induction therapy and repeated chemotherapy after recurrence, allows long-term survival even in such complex cases.
